# TaqMan MGB Probe Fluorescence Real-Time Quantitative PCR for Rapid Detection of Chinese Sacbrood Virus

**DOI:** 10.1371/journal.pone.0052670

**Published:** 2013-02-08

**Authors:** Ma Mingxiao, Liu Jinhua, Song Yingjin, Li Li, Li Yongfei

**Affiliations:** 1 Department of Laboratory Animal Center, Liaoning Medical University, Jinzhou, China; 2 Jilin Entry-Exit Inspection and Quarantine Bureau, Changchun, China; 3 Agriculture and Biology Engineering College, Tianjin University, Tianjin, China; Temple University School of Medicine, United States of America

## Abstract

Sacbrood virus (SBV) is a picorna-like virus that affects honey bees (*Apis mellifera*) and results in the death of the larvae. Several procedures are available to detect Chinese SBV (CSBV) in clinical samples, but not to estimate the level of CSBV infection. The aim of this study was develop an assay for rapid detection and quantification of this virus. Primers and probes were designed that were specific for CSBV structural protein genes. A *Taq*Man minor groove binder (MGB) probe-based, fluorescence real-time quantitative PCR was established. The specificity, sensitivity and stability of the assay were assessed; specificity was high and there were no cross-reactivity with healthy larvae or other bee viruses. The assay was applied to detect CSBV in 37 clinical samples and its efficiency was compared with clinical diagnosis, electron microscopy observation, and conventional RT-PCR. The *Taq*Man MGB-based probe fluorescence real-time quantitative PCR for CSBV was more sensitive than other methods tested. This assay was a reliable, fast, and sensitive method that was used successfully to detect CSBV in clinical samples. The technology can provide a useful tool for rapid detection of CSBV. This study has established a useful protocol for CSBV testing, epidemiological investigation, and development of animal models.

## Introduction

Sacbrood virus (SBV) is a picorna-like virus that affects the honey bee (Apis mellifera) and results in the death of the larvae [1]. Larvae that have been infected with SBV fail to pupate and ecdysial fluid, rich in SBV, accumulates beneath their unshed skin. Infected larvae change in color from white to pale yellow and then die. Shortly after death they dry out and form a distinctive dark brown gondola-shaped scale [2]. SBV causes a fatal infection in bee larvae, but may also infect the adult bee and infected workers may have decreased life spans [3], [4]. When this infection occurs in adults, however, obvious physical signs of disease are lacking [3], [5]SBV infection occurs most frequently in the spring and this timing is believed to reflect the availability of susceptible larvae and young adults that are at their height in this season when the colony is growing most rapidly [3].

The SBV that infects the Chinese honeybee was named Chinese SBV (CSBV). CSBV was first described in Guangdong China in 1972, and re-emerged in Liaoning China in 2008 [6], when it caused lethal disease in individual bees or the collapse of entire colonies.

Several procedures have been utilized previously to detect CSBV. These methods include reverse transcriptase polymerase chain reaction (RT-PCR) [6], [7] and loop-mediated isothermal amplification (LAMP) assay [8]. Although these techniques can be used to characterize CSBV in clinical samples, they cannot estimate the level of CSBV infection. The quantitation of CSBV would permit a better understanding of virus infection, both in individual bees and in the hive.

Real-time RT-PCR detection methods have been developed recently for the detection and quantitation of bee viruses [9]–[13]. This paper describes a TaqMan MGB (minor groove binder) probe fluorescence real-time quantitative PCR assay to quantify CSBV. Standard curves from a plasmid that contained a partial sequence of the CSBV genome were used to obtain an absolute quantitation of CSBV. The specificity, sensitivity and stability of the method were validated from a standard DNA curve. The established TaqMan MGB probe fluorescence real-time quantitative PCR assay was applied to detect CSBV in 37 clinic samples, and its efficiency was compared with that of clinic diagnosis, electron microscopy observation, and conventional RT-PCR assay.

## Materials and Methods

### Sample preparation, RNA extraction and cDNA synthesis

CSBV (CSBV-LNQY) was obtained from a natural outbreak in Liaoning China. Infected larvae/pupae were stored at –70°C until required. Viral genomic RNA was extracted from the purified virus preparation using TRIzol reagent (Invitrogen, Carlsbad, California, USA). For purification of the virus, CSBV-infected larvae were homogenized in 5 ml NT buffer (100 mM NaCl, 10 mM Tris pH 7.4) and the macerate was clarified at 1000 *g* for 10 min. The supernatant was extracted with an equal volume of 1,1,2-trichlorotrifluoroethane and the aqueous phase was layered over a discontinuous CsCl gradient (1.5 g/cm^3^ and 1.2 g/cm^3^) and centrifuged at 270 000 *g* for 1 h in an SW50 rotor. The material at the CsCl interface was harvested and adjusted to a volume of 5 ml (final density 1.38 g/cm^3^) with CsCl solution and centrifuged at 270 000 *g* overnight. Two light-scattering bands were formed that were collected separately and diluted with NT buffer; virus was collected by sedimentation at 270 000 *g* for 1 h. Pellets were resuspended in NT buffer. Complementary DNA (cDNA) was synthesized using 12.5 μl of eluted RNA, oligo(dT)18 primer [14] and avian myeloblastosis virus (AMV) reverse transcriptase XL (TaKaRa, Dalian, China) according to the manufacturer's instructions.

### Real-time quantitative PCR protocol by TaqMan MGB probe assay

Primer pairs and probes specific for CSBV were designed based on the nucleotide sequences of CSBV published previously in the GenBank database (accession no. AF469603 and HM237361.1). The primers and probes used for *Taq*Man MGB probe fluorescence real-time quantitative PCR were designed using the Primer5 software. The sequence of the forward primer is 5′-CCTGGGAAGTTTGCTAGTATTTACG-3′ and of the reverse primer is 5′-CCTATCACATCCATCTGGGTCAG-3′. The *Taq*Man MGB probe sequence was 5′- CGACATACCCGCAAATTCAGCACGC-3′, which was labeled with the fluorescent reporter dye FAM (6-carboxyfluorescein) at the 5′ end and with the fluorescent NFQ-MGB at the 3′ end. A 161-bp product was expected when these primers were used. The primers and the *Taq*Man MGB probes were synthesized and labeled by TaKaRa, Dalian, China.

TransStart™ Probe qPCR SuperMix (Beijing TransGen Biotech Co., Ltd.) was used for amplification by *Taq*Man PCR. The PCR reaction mixture contained 500 nM of each primer, 250 nM of the probe, 2× PCR buffer 12.5 μl, Reference 0.5 μl and 2 μl of standard template plasmid or cDNA in a 25 μl total reaction volume. The thermal cycling conditions were 30 sec at 95°C, followed by 40 cycles that consisted of a denaturation step at 95°C for 5 sec, annealing and extension step at 60°C for 60 sec. The ABI Prism 7000 system (Applied Biosystems ABI) was used for amplification and detection.

### Preparation of the plasmid DNA standard for calibration of the CSBV TaqMan MGB probe PCR assay

A standard DNA curve was generated with a 3.01 kb plasmid that was obtained by cloning a 320-bp PCR fragment located in the VP1 gene of CSBV into the pMD® 18-T Vector (TaKaRa, Dalian, China.). The plasmid was quantified based on the DNA concentration determined by UV (ultraviolet) light spectrometry; stock solutions were prepared in Tris–EDTA (TE) buffer that ranged in concentration from 5×10^8^ to 5 DNA copies per microliter. A standard DNA curve for the range of 5×10^8^ to 5×10^3^ DNA copies per reaction was generated by analysing 2 μl of each dilution in triplicate by *Taq*Man PCR.

### Evaluation of the method

The limit of detection of the CSBV *Taq*Man PCR was determined by serial 10-fold dilution of the DNA standard prepared in triplicate.

The specificity of the CSBV *Taq*Man PCR was assessed by testing cDNAs generated from deformed wing virus (DWV), black queen cell virus (BQCV), acute bee paralysis virus (ABPV), and chronic bee paralysis virus (CBPV) isolates, which were provided by Professor Laurent Gauthier, healthy larvae and two positive samples were included as controls for the specific RT-PCR tests.

The reproducibility of the CSBV *Taq*Man PCR assay was demonstrated by evaluation of the variability of the C_T_ values obtained after amplification of 10-fold serial dilutions of the plasmid DNA standard ranging from 10^2^ to 10^6^ copies per reaction in triplicate during the same experiment.

### Validation of the CSBV TaqMan PCR method on clinical samples

Thirty-seven clinical samples collected from Qingyuan, Jinzhou, Tieling, Yingkou, Dalian and Liaozhong areas of Liaoning Province and Huinan Ji'an areas of Jilin Province, China from 2008 to 2011 were tested to determine the feasibility of the *Taq*Man PCR assay. At the same time, all mentioned samples were subjected to conventional RT-PCR and electron microscopy.

## Results

### Standardisation of the CSBV TaqMan PCR assay

**Figure 1:**
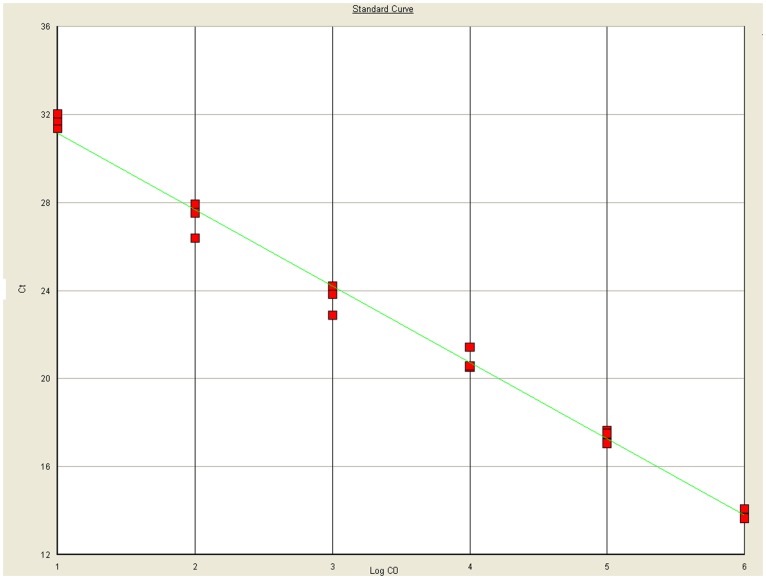
Standard curve of *Taq*Man MGB probe real-time quantitative polymerase chain reaction (PCR) for Chinese sacbrood virus (CSBV). DNA standard curves of CSBV *Taq*Man PCR assay using a FAM-MGB-labeled *Taq*Man probe obtained with a 10-fold serial dilution (5×10^8^ to 5×10^3^ DNA copies) of a 3.1-kb plasmid that included a 320-bp fragment located in the CSBV VP1 gene. The standard curve was obtained by linear regression analysis of the C_T_ measured for each amplification (y-axis) vs. the log copy number for each standard dilution (x-axis). The slope of the standard curve (–3.47) and the correlation coefficient are indicated *r* ^2^ = 0.99 (Y = –3.47X+34.60). The red box represents Ct.

The assay was calibrated using a plasmid DNA control as a standard. The standard curve generated from the amplification plot of the 10-fold serial dilution experiment (Fig. 1) showed a linear correlation between C_T_ values and the DNA load over a 6-log range (*r* ^2^ = 0.99). The slope of the DNA standard curve was 3.47, which indicated that the efficiency of the CSBV *Taq*Man PCR was 95%.

**Figure 2:**
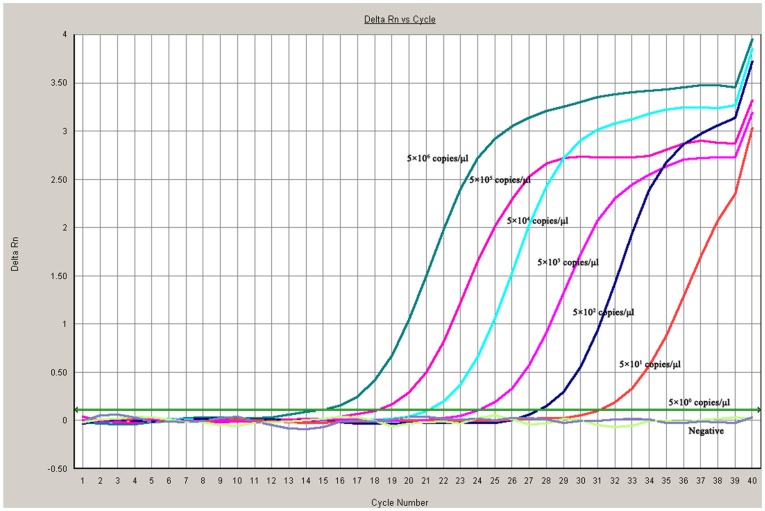
Sensitivity of the *Taq*Man MGB probe real-time quantitative polymerase chain reaction (PCR) for Chinese sacbrood virus (CSBV). To evaluate the sensitivity of the *Taq*Man MGB probe real-time RT-PCR assay. Amplification of seven 10-fold dilutions of DNA plasmid gave a titer that ranged from 5×10^6^ to 5×10^0^ DNA copies per reaction mixture. The reactions were performed in triplicate. The limit of detection of the *Taq*Man MGB probe real-time PCR was 50 CSBV genome equivalent copies.

The limit of detection of the CSBV *Taq*Man PCR was 50 CSBV genome equivalent copies (Fig. 2); this result compared favourably with conventional PCR for which the limit of detection of CSBV genome from plasmid DNA control was 5×10^3^ copies (data not shown).

### Specificity and reproducibility of the CSBV TaqMan PCR

**Figure 3:**
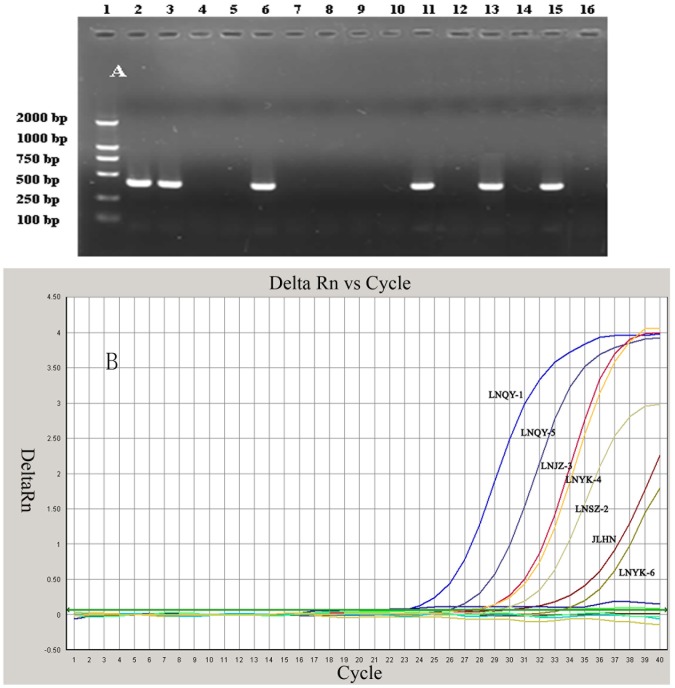
Comparison of TaqMan PCR assay and reverse transcriptase polymerase chain reaction (RT-PCR) methods by testing collected clinical samples. The 16 collected clinical samples were tested using the TaqMan RT-PCR and RT-PCR, respectively. (A) RT-PCR test results. (B) TaqMan RT-PCR test results. The LNQY-1, LNJZ-3, LNYK-4, JLHN, LNQY-5 and LNSZ-2 samples tested positive using conventional RT-PCR and the TaqMan RT-PCR method, but the LNYK-6 samples tested positive using the TaqMan RT-PCR method, however the samples were negative by electron microscopy and conventional RT-PCR. lane 1. DNA marker DL2000, lane 2. LNQY-1, lane 3. LNQY-5, lane 4. LNQY-2, lane 5. LNYK-3, lane 6. LNYK-4, lane 7. LNYK-6, lane 8. LNLZ-1, lane 9. LNLZ-3, lane 10 JLJA, lane 11. JLHN, lane 12. LNJZ-1, lane 13. LNJZ-3, lane 14 LNJZ-5, lane 15 LNSZ-2 lane 16. JLBC.

CBPV specificity was confirmed by a BLAST search of the amplicon (116 bp) generated by *Taq*Man PCR. No significant similarity was found. Furthermore, no amplification was detected when these *Taq*Man PCR conditions were performed on cDNAs obtained from CBPV, ABPV, BQCV or DWV samples (Fig. 3). The coefficient of variation (CV) of the mean C_T_ values obtained from the DNA standard curve ranged from 0.27 to 1.4% (Table 1).

**Table 1:** Reproducibility of *Taq*Man minor groove binder (MGB) probe fluorescence real-time quantitative polymerase chain reaction (PCR).

No. of CSBV copies (amount of cDNA per mix)	MGB-labelled CSBV probe	
	C_T_ value	CV (%)
5×10^6^ copies/μl	14.90±0.03383	0.39
5×10^5^ copies/μl	17.95±0.04509	0.44
5×10^4^ copies/μl	21.26±0.1715	1.4
5×10^3^ copies/μl	24.96±0.08083	0.57
5×10^2^ copies/μl	27.63±0.04055	0.27
5×101 copies/μl	31.15±0.09045	0.73

The reproducibility of the Chinese sacbrood virus (CSBV) *Taq*Man PCR assay was demonstrated by evaluating the variability of the C_T_ values obtained after amplification of 10-fold serial dilutions of the plasmid DNA standard ranging from 10^1^ to 10^6^ copies per reaction in triplicate during the same experiment. The coefficient of variation (CV) of the mean C_T_ values obtained for the DNA standard curve ranged from 0.27 to 1.4%.

### Validation of the CSBV TaqMan PCR method on clinical samples

**Figure 4:**
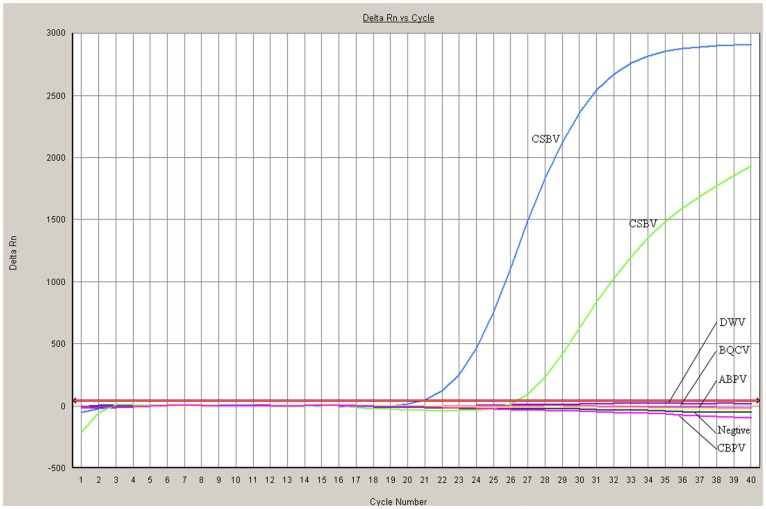
Comparison of the specificity of Chinese sacbrood virus (CSBV) *Taq*Man PCR assay for CSBV detection. Chinese sacbrood virus strains gave a positive reaction using the *Taq*Man PCR assay method, no cross-reactivity with other clinically related viruses, including deformed wing virus (DWV), black queen cell virus (BQCV), acute been paralysis virus (ABPV), and chronic bee paralysis virus (CBPV), was detected.

The 37 collected clinical samples were tested to determine the feasibility of the TaqMan PCR assay. At the same time, all mentioned samples were subjected to conventional RT-PCR and electron microscopy. As shown in Table 2 (Fig. 3), the LNQY-1, LNJZ-3, LNYK-4 and JLHN samples presented severe signs of sacbrood disease and all three methods yielded positive results. The LNQY-5 and LNSZ-2 samples tested positive using conventional RT-PCR and the TaqMan RT-PCR method, however the samples were negative by electron microscopy and for clinical symptoms, The LNYK-6 samples tested positive using the TaqMan RT-PCR method, however the samples were negative by electron microscopy and conventional RT-PCR (Table 2). Furthermore, DWV, BQCV, ABPV and CBPV were also detected by RT-PCR, but these viruses were not detected by other means (Fig. 4).

**Table 2:** Detection of 37 Chinese sacbrood virus (CSBV) clinical samples: of *Taq*Man minor groove binder (MGB) probe fluorescence real-time quantitative PCR, reverse transcriptase polymerase chain reaction (RT-PCR) and electron microscopy methods.

Samples	Area of collecting sample	RT-PCR	*Taq*Man MGB	CSBV copies	EM
LNQY-1	Qingyuan	+	+	3.59×10^5^ copies/μl	+
LNQY-2	Qingyuan	–	–		–
LNQY-3	Qingyuan	–	–		–
LNQY-4	Qingyuan	–	–		–
LNQY-5	Qingyuan	+	+		–
LNQY-6	Qingyuan	–	–		–
LNJZ-1	Jinzhou	–	–		–
LNJZ-2	Jinzhou	–	–		–
LNJZ-3	Jinzhou	+	+		+
LNJZ-4	Jinzhou	–	–		–
LNJZ-5	Jinzhou	–	–		–
LNTL-1	Tieling	–	–		–
LNTL-2	Tieling	–	–		–
LNTL-3	Tieling	–	–		–
LNTL-4	Tieling	–	–		–
LNTL-5	Tieling	–	–		–
LNYK-1	Yingkong	–	–		–
LNYK-2	Yingkong	–	–		–
LNYK-3	Yingkong	–	–		–
LNYK-4	Yingkong	+	+		+
LNYK-5	Yingkong	–	–		–
LNYK-6	Yingkong	–	+		–
LNDL-1	Dalian	–	–		–
LNDL-2	Dalian	–	–		–
LNDL-3	Dalian	–	–		–
LNDL-4	Dalian	–	–		–
LNDL-5	Dalian	–	–		–
LNLZ-1	Liaozhong	–	–		–
LNLZ-2	Liaozhong	–	–		–
LNLZ-3	Liaozhong	–	–		–
LNLZ-4	Liaozhong	–	–		–
LNLZ-5	Liaozhong	–	–		–
LNSZ-1	Suizhong	–	–		–
LNSZ-2	Suizhong	+	+		–
JLHN	Huinan	+	+		+
JLJA	Jian	–	–		–
JLBC	Baicheng	–	–		–

## Discussion

Quantitative real-time polymerase chain reaction (qRT-PCR), is a laboratory technique based on PCR that is used to amplify and simultaneously quantify a targeted DNA molecule. The standard DNA probes are labeled at the 5′ end with a fluorochrome reporter (usually 6-carboxyfluorescein [6-FAM]) and a fluorochrome quencher (6-carboxy-tetramethyl-rhodamine [TAMRA]) at the 3′ end. A new technology of qRT-PCR that uses MGB probes [15] for fluorescence quantitative PCR, has been developed recently for the detection and quantitation DNA molecules. The DNA probes with the MGB groups form extremely stable duplexes with single-stranded DNA targets, and therefore allow shorter probes to be used for hybridization-based assays. MGB probes have higher melting temperature (Tm) and increased specificity when compared with unmodified DNA, especially when a mismatch occurs in the MGB region of the duplex. The fluorogenic MGB probes were more specific for single-base mismatches and fluorescence quenching was more efficient, and therefore gave increased sensitivity. In summary, MGB probes were more sequence specific than standard DNA probes, especially for single-base mismatches at elevated hybridization temperatures. In this paper, a *Taq*Man MGB probe fluorescence real-time quantitative PCR assay was developed for the detection and quantitation of CSBV. Kerstin Wernike [16] describe a *Taq*Man MGB probe fluorescence real-time quantitative PCR assay for the detection of SBV, Howere the *Taq*Man MGB hasn't been evaluated. This paper is the first to describe a *Taq*Man MGB probe fluorescence real-time quantitative PCR assay for the especially detection and quantitation of CSBV in honey bees.

The *Taq*Man MGB probe with primers specific for the CSBV VP1 gene was used. The standard curve generated with the plasmid that contained a partial sequence of the CSBV genome showed that quantitation of this genome was linear over six orders of magnitude. The efficiency of the standard curve and its good correlation was confirmed. Quantitation of the positive control from CSBV-infected larvae gave similar results for both methods compared to the values obtained by UV spectrometry. These results validated the use of the DNA standard curve to quantify CSBV in bee samples. The limit of detection of this *Taq*Man PCR method was 50 CSBV genome equivalent copy numbers, which represented an improvement in the limit of detection by thr conventional the PCR method developed previously in our laboratory [5], for which the limit of detection was 5×10^3^ CSBV copies. The reproducibility of several experiments showed the high reproducibility of the method for the standard curve (0.27 to 1.4%), and for the efficiency of cDNA synthesis from the positive control (0.95%). The feasibility of the *Taq*Man PCR assay was validated by detection of 37 clinical samples using conventional PCR and the *Taq*Man PCR. The *Taq*Man PCR assay was more sensitivity than conventional PCR.

In summary, in this paper we provide a quantitative description of CSBV infection in larvae in China. Our data demonstrated that real-time quantitative RT-PCR is a specific, sensitive, robust, and reproducible assay with practical applications in the diagnosis of honey bee virus diseases and analysis of virus infection.
